# The complete chloroplast genome sequence of rare and endangered *Camellia pubipetala* Y. Wan & S. Z. Huang (Theaceae) of South China

**DOI:** 10.1080/23802359.2021.1915207

**Published:** 2021-09-13

**Authors:** Yuan-yuan Fan, Xiao-xia Ye, Min Wei, Bo Li, Yu-lin Zhu, Zhou Xing-wen

**Affiliations:** a College of Pharmacy, Guilin Medical University, Guilin, PR China; b Guangxi Key Laboratory of Agricultural Resources Chemistry and Biotechnology, Yulin Normal University, Yulin, PR China

**Keywords:** *Camellia pubipetala* Y.Wan & S.Z.Huang (Theaceae), complete chloroplast genome, phylogeny tree

## Abstract

The complete chloroplast genome sequence of rare and endangered *Camellia pubipetala* Y. Wan & S. Z. Huang (Theaceae) was mentioned in this research. By studying comparatively, we found that the *C. pubipetala* Y. Wan & S. Z. Huang chloroplast genome was 156,993 bp in length and composed of 86,590 bp LSC, 18,211 bp SSC, and two reverse repeating regions with 26,090 bp. The whole GC content was 37.33%. The genome encoded 116 functional genes, including 80 protein-coding genes, 32 tRNA genes, and 4 rRNA genes. In order to find the phylogenetic relationship of *C. pubipetala* Y. Wan & S. Z. Huan*g* within *Camellia* genus, we reconstructed phylogenetic tree. The results indicate that *C. pubipetala* Y. Wan & S. Z. Huang was closely related to *Camellia huana voucher* and *Camellia ptilosperma.*

*Camellia pubipetala* Y. Wan & S. Z. Huang belongs to a species in the genus *Camellia* (family Theaceae). It only distributes in several small areas of Longan and Daxin, southwestern Guangxi, China and grows in evergreen forests on limestone (Hu et al. 2019). Because of its beautiful appearance, it has ornamental value. In addition to this, it has high medicinal value and Edible value (He et al. 2016). Due to the narrow distribution area and the damage of ecological environment from human being, the survival of these species is threatened and the number of wild population decreases sharply. The species are on the verge of extinction (Qin et al. 2017). Until now, the issue of phylogenetic relationship among Camellia species has failed to address. The study of chloroplast genome is conducive to solve the evolutionary position and protect the germplasm resources of *C. pubipetala* Y. Wan & S. Z. Huang (Liu et al. 2012; Xia et al. 2017).

The healthy and fresh leaves of *C. pubipetala* Y. Wan & S. Z. Huang were collected from a tree and came from Longan County, Guangxi, China (107.68°E longitude and 23.18°N latitude), and dried rapidly through silica gel. The certificate specimens were deposited in the Herbarium of Yulin Normal University. The leaves were separated for use in this experimental research. In order to get cleaner and more complete chloroplast genomic DNA, we extracted total genomic DNA from the samples (∼100 mg) by using the modified CTAB method. Then it was sent to Wuhan Bena Biotechnology Co., Ltd in order to build cpDNA library, after then, using Illumina HiSeq 4000 sequencing platform (Illumina, San Diego, CA) for sequencing. After filtering the raw data by removing the connector sequence and low-quality reading area, we obtained at least 3.83 Gb clean data of *C. pubipetala* Y. Wan & S. Z. Huang with the help of CLC Cenomics Workbench version 7.5 software (CLC Bio, Aarhus, Denmark) (Zhang et al. 2019). Then, the contigs were aligned to the reference species (*Camellia petelotii* NC_024661) and get the relative location of the contig sequences, thereby *C. pubipetala* Y. Wan & S. Z. Huang was compared and corrected. The complete chloroplast genome of *C. pubipetala* Y. Wan & S. Z. Huang was preliminarily annotated using GENEIOUS version 11.1.5 software (Biomatters Ltd., Auckland, New Zealand), followed by manual correction. Finally, the corresponding position of the genes was adjusted according to the start codon and the stop codon. Meanwhile, the whole data submitted to the NCBI database to obtain the genome entry sequence number.

The total chloroplast genome of *C. pubipetala* Y. Wan & S. Z. Huang was 156,993 bp in length and composed of 86,590 bp LSC, 18,211 bp SSC, and 2 reverse repeating regions with 26,090 bp. The whole GC content was 37.33%. The genome encoded 116 functional genes, including 80 protein-coding genes, 32 tRNA genes, and 4 rRNA genes.

Furthermore, we downloaded 35 complete Camellia chloroplast genomes from NCBI, according to the whole chloroplast genome of *C. pubipetala* Y. Wan & S. Z. Huang, the phylogenetic tree of Camellia was constructed by using maximum likelihood (ML) analysis with RAxML version 8.2.4 (Stamatakis, 2004; Figure 1). The consequence indicated that all the Camellia species formed a monophyletic branch. We found that *C. pubipetala* Y. Wan & S. Z. Huang and *Camellia huana voucher* formed a clade with high bootstrap support (100%), and then shared a sister relationship with *Camellia ptilosperma*. This result supports the suggestion that *C. sinensis* var. *assamica* was grouped with *C. grandibracteata* with 100% bootstrap support based on nrDNA ITS (Zhang et al. 2019).

**Figure 1.**
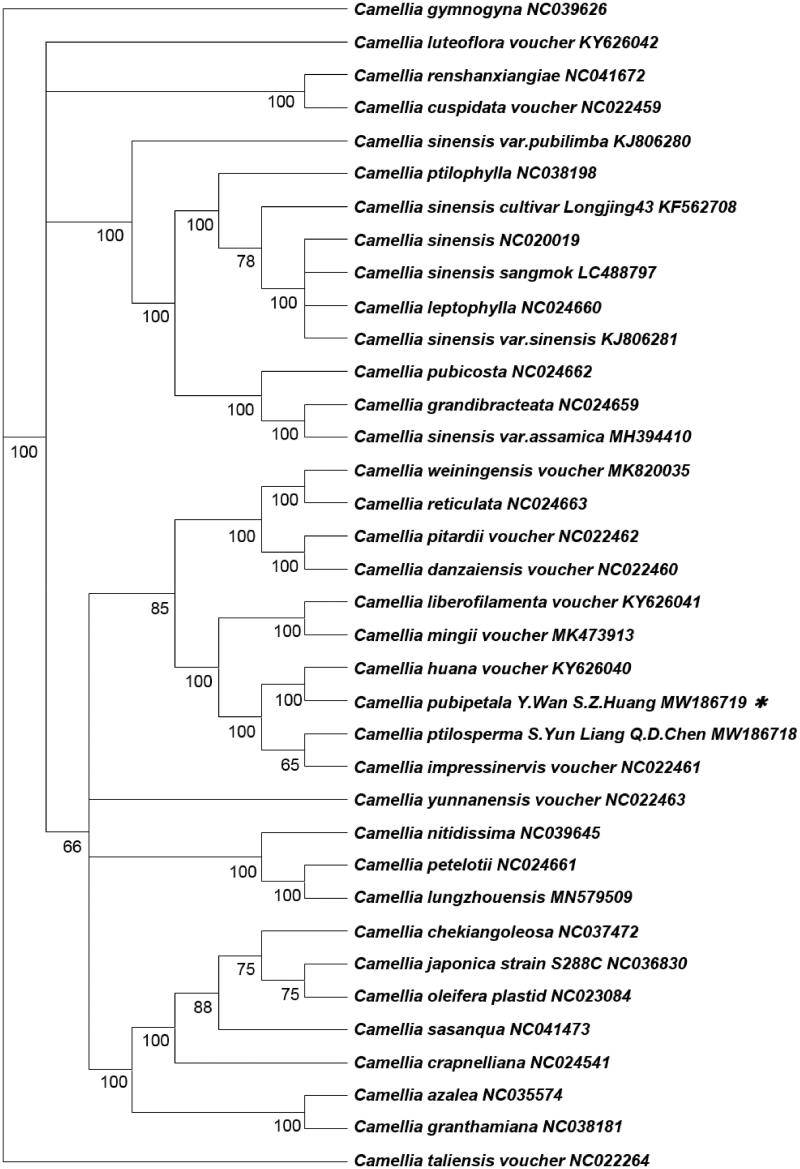
Based on chloroplast genome sequences, we used by maximum likelihood (ML) analysis to reconstruct phylogenetic tree, containing *Camellia pubipetala* Y. Wan & S. Z. Huang * sequence in this findings. Numbers below or above branches are assessed by ML bootstrap.

## Data Availability

The data that support the findings of this study are openly available in GenBank of NCBI at https://www.ncbi.nlm.nih.gov, reference number MW186719.
